# Nephrologists’ Perspectives on Gender Disparities in CKD and Dialysis

**DOI:** 10.1016/j.ekir.2021.10.022

**Published:** 2021-11-09

**Authors:** Allison Tong, Nicole Evangelidis, Amelie Kurnikowski, Michal Lewandowski, Philipp Bretschneider, Rainer Oberbauer, Amanda Baumgart, Nicole Scholes-Robertson, Tanja Stamm, Juan Jesus Carrero, Roberto Pecoits-Filho, Manfred Hecking

**Affiliations:** 1 Clinical Division of Nephrology and Dialysis, Department of Internal Medicine III, Medical University of Vienna, Vienna, Austria; 2 Centre for Kidney Research, The Children’s Hospital at Westmead, Westmead, Australia; 3 Sydney School of Public Health, The University of Sydney, Sydney, Australia; 4 Center for Medical Statistics, Informatics and Intelligent Systems, Medical University of Vienna, Vienna, Austria; 5 Department of Medical Epidemiology and Biostatistics, Karolinska Institutet, Stockholm, Sweden; 6 School of Medicine, Pontifícia Universidade Católica do Paraná, Curitiba, Brazil; 7 Arbor Research Collaborative for Health, Ann Arbor, Michigan, USA

**Keywords:** chronic kidney disease, disparities, gendering, kidney replacement therapy initiation, nephrologists, perspectives

## Abstract

**Introduction:**

Globally, there are more women with chronic kidney disease (CKD), yet they comprise only 40% of patients receiving kidney replacement therapy by dialysis. We aimed to describe the perspectives of nephrologists on gender disparities in access to care and outcomes in CKD and dialysis.

**Methods:**

We conducted semistructured interviews with 51 nephrologists (28, 55% women) from 22 countries from October 2019 to April 2020. Transcripts were analyzed thematically.

**Results:**

We identified 6 themes. Related to women were primary commitment to caregiving (with subthemes of coordinating care, taking charge of health management, deprioritizing own health, centrality of family in decision-making); vigilance and self-reliance (diligence and conscientiousness, stoicism and tolerating symptoms, avoiding burden on family, isolation and coping alone); and stereotyping, stigma, and judgment (body image, dismissed as anxiety, shame and embarrassment, weakness and frailty). Related to men was protecting masculinity (safeguarding the provider role, clinging to control, self-regard, and entitled). Decisional power and ownership included men’s dominance in decision-making and women’s analytical approach in treatment decisions. Inequities compounded by social disadvantage (financial and transport barriers, without social security, limited literacy, entrenched discrimination, vulnerability) were barriers to care for women, particularly in socioeconomically disadvantaged communities.

**Conclusion:**

Nephrologists perceived that women with CKD faced many challenges in accessing care related to social norms and roles of caregiving responsibilities, disempowerment, lack of support, stereotyping by clinicians, and entrenched social and economic disadvantage. Addressing power differences, challenging systemic patriarchy, and managing unconscious bias may help to improve equitable care and outcomes for all people with CKD.

## Graphical abstract

**Figure:**
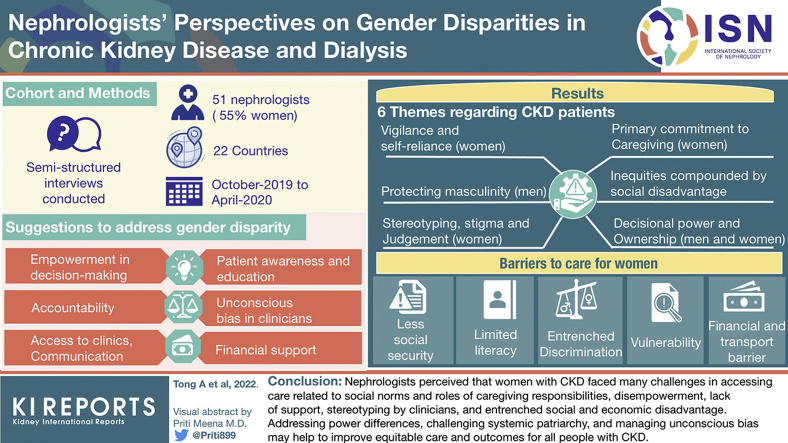

See Commentary on Page 375

Globally, there are more women with CKD compared with men, yet women comprise approximately 40% of patients receiving kidney replacement therapy.^1^ Late referrals for kidney replacement therapy and delayed initiation of dialysis are more common in women.^2^^,^^3^ Men with CKD have an increased risk of progression to kidney failure requiring kidney replacement therapy and death compared with women.^4^ In the dialysis population, women have a higher risk of morbidity, hospitalization, and impaired quality of life and are more likely to withdraw from dialysis than men.^5^, ^6^, ^7^

These differences may be due to biology and pathophysiology of CKD, estimations of kidney function (e.g., diagnosis based on estimated glomerular filtration rate), dialysis adequacy, and the potential effect of hormones and pregnancy on kidney function.^10^, ^11^, ^8^, ^9^ Another reason relates to gender-based inequities in access to health care. Awareness of CKD is lower in women compared with men,^12^, ^13^, ^14^ and there is also concern that sociocultural discrimination and disadvantage are major barriers to care (including dialysis) for women with CKD.^11^ Existing studies have predominantly focused on access to transplantation^15^ and racial and socioeconomic disparities^16^, ^17^, ^18^; however, little remains known on gender disparities in access and outcomes in CKD and dialysis.^3^^,^^4^^,^^11^^,^^19^

According to the World Health Organization, gender refers to the “roles, behaviours, activities, attributes and opportunities that any society considers appropriate for women and men,”^20^ whereas sex refers to the biological attributes, including anatomical, endocrine, or genetic traits. Gender disparities refer to differences in women’s and men’s access to health care and outcomes, and it is recognized that women encounter more barriers related to gender because of patriarchal cultures, family responsibilities, and economic dependence.^20^, ^21^, ^22^ The aims of this study were to describe the perspectives of nephrologists on gender disparities in CKD and dialysis, to inform ways to address gender disparities, and to improve equitable access, care, and outcomes in patients with CKD.

## Methods

We used the Consolidated Criteria for Reporting Qualitative Health Research.^23^

### Participant Selection

Practicing nephrologists were eligible to participate. The nephrologists were purposively selected to include a diverse range of age, gender, years of clinical experience, and location (including low-, middle-, and high-income countries). The nephrologists were identified through our collegial networks and invited by e-mail, and participants could also nominate other colleagues to participate. The University of Sydney approved this study (2019-899).

### Data Collection

The interview guide was informed by gender theories,^20^^,^^24^, ^25^, ^26^, ^27^ literature on gender disparities in CKD,^1^^,^^19^^,^^28^^,^^29^ and discussion among the investigator team (Supplementary File 1). Authors AT, NE, AK, and PB conducted semistructured interviews in person (at conference centers, clinic) and by zoom video conference from October 2019 to April 2020, in English or German language, until data saturation, that is, when little or no new concept, was identified in subsequent interviews. The interviews were recorded and transcribed, and where required translated into English.

### Data Analysis

Using thematic analysis, author AT conducted line-by-line coding of the transcripts and inductively identified preliminary concepts on gender disparities in CKD and dialysis. Of note, concepts on biological differences were outside the scope of the current study. Similar concepts were grouped into themes, and patterns were identified among the themes. The themes were discussed among the investigators and revised until it was agreed that the analysis captured the full range and depth of the data obtained. AT coded the transcripts to the agreed themes using HyperRESEARCH software (ResearchWare, Inc., Randolph, MA; version 3.3). We conducted member checking, whereby the preliminary findings were sent to participants to provide comments, and we integrated any additional perspectives in the final analysis.

## Results

Of the 56 invited nephrologists, 51 (91%) from 36 centers across 22 countries participated (Table 1). There were 4 who did not respond (no reason provided) and 1 who declined owing to illness. Of the participants, 28 (55%) were women, 30 (59%) were from countries in which English was not an official language, and 9 (18%) were from low- and middle-income countries. The average duration of the interviews was 35 minutes, and 25 (49%) were conducted in person.

**Table 1:** Participant characteristics (*N* = 51)

Characteristics	N	%
Sex		
Women	28	55
Men	23	45
Age group (yr)		
20–29	1	2
30–39	9	18
40–49	11	22
50–59	16	31
60–69	10	20
70+	4	8
Ethnicity		
White	28	55
Asian	10	20
Hispanic	7	14
African	4	8
African American	1	2
Middle Eastern	1	2
Years of clinical experience in nephrology		
<5	2	4
5–10	8	16
11–20	15	29
21–30	9	18
31–40	16	31
40+	1	2
Number of patients with CKD (not on KRT)		
0	1	2
51–100	2	4
101–300	5	10
301–500	7	14
501–1000	5	10
1001–2000	11	22
2000+	14	27
Number of patients on hemodialysis		
1–50	6	12
51–100	10	20
101–200	17	33
201–300	6	12
301–400	2	4
400+	9	18
Number of patients on peritoneal dialysis		
0	2	4
1–50	20	39
51–100	14	27
101–200	7	14
201–300	5	10
Country^a^		
Austria	11	22
Australia	8	16
United States	7	14
Belgium	2	4
Chile	2	4
Mexico	2	4
New Zealand	2	4
Nigeria	2	4
South Africa	2	4

CKD, chronic kidney disease; KRT, kidney replacement therapy.

a 1 participant each from Brazil, Canada, China (Hong Kong), France, India, Italy, Japan, Malaysia, Poland, Sweden, Switzerland, United Kingdom, and Uruguay.

**Figure 1:**
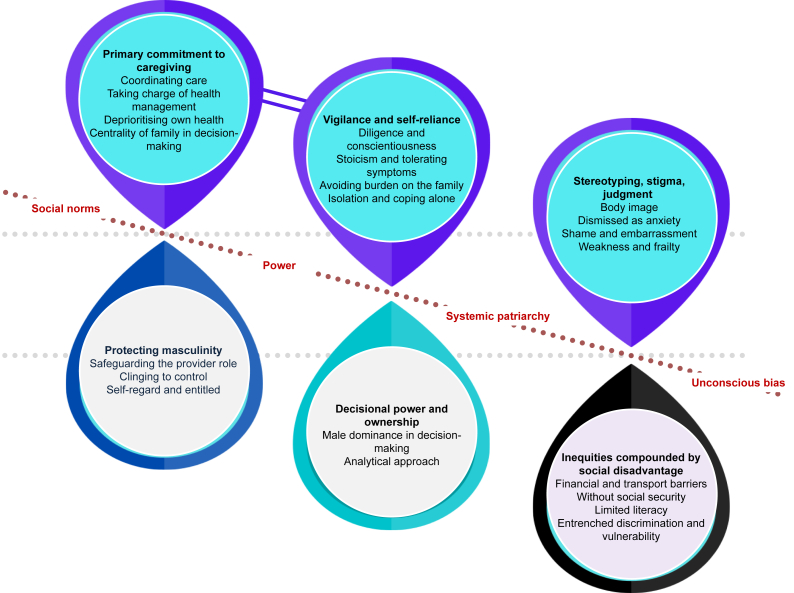
Thematic schema.

We identified the following 6 themes: primary commitment to caregiving; vigilance and self-reliance; stereotyping, stigma, and judgment; protecting masculinity; decisional power and ownership; and inequities compounded by social disadvantage. The respective subthemes are described subsequently with supporting quotations provided in Table 2. Data that are in reference to a specific gender (including both participants and patients) or health care setting are indicated accordingly. Furthermore, given the explicit focus on the perceived reasons for gender disparities, statements indicating participants did not perceive any differences were not described in detail. A thematic schema illustrating patterns among themes is found in Figure 1.

**Table 2:** Supporting quotations

Theme	Quotations
Primary commitment to caregiving	
Coordinating care	The spouse is an active partner in the shared decision making, she’s an active partner in making sure they get to CKD classes and education. (04, woman) The wife plays a very important role in supporting the patient. I’d have had patients on dialysis for 13 years and in fact they were alive because of their wives. (09, man) Women more frequently go with men, go with them to the outpatient clinic, […] and that is not so for men that they probably don’t go as much if the patient is women. (13, woman)
Taking charge of health management	She will look after the man but not of herself. (02, woman) Some male patients don’t know what pills they take. The only one that knows this is the wife. (13, woman) The wives may help with their dialysis, […] for the home hemo patients that I have who are male patients. These male patients really fall apart when their female partners become sick […]. (22, woman)
Deprioritizing own health	Women forget that they need to take care of themselves first. (04, woman) She would miss out on at least once a week dialysis session because she felt that she had to be at home to run the errands and etc. for the young children. (22, woman) For the mother to leave the child without another carer available and come to the clinic is a big deal. That might result in presenting later as they otherwise would. (43, man) Women might be sometimes a little bit disadvantaged just because they’re the ones left at home with the children and don’t have time then to focus on themselves […]. (45, woman)
Centrality of family in decision-making	The woman’s goals. Many of them do involve goals that goes beyond them as a person. Their goals, a lot of the time, involves being the caring grandmother or the caring spouse or this and that. (04, woman) She’s more passive. It’ll be more about what’s right for that family unit. (14, man) If say childcare or getting kids to school is an issue then that’s something we’ll discuss. It comes up more often with women. (15, woman)
Vigilance and self-reliance	
Diligence and conscientiousness	The females are more likely to be the ones who are there every day, and same thing with like clinic visits, they tend to show up more regularly for appointments. (08, man) Women go to the outpatient clinics more frequently than men. Women tend to go to the doctor more frequently and perhaps to be diagnosed earlier and just to go more frequently. (13, woman) Perhaps women are more likely to submit to the doctors or tend to believe them more. Men are perhaps more stubborn. (32, man)
Stoicism in tolerating symptoms	Women feel like […] all the other responsibilities take priority. Sometime they just get so adapted to like really poor quality of life […] pushing through all this while men are not going to do that. (04, woman) It’s socialization. Women are less likely to shout “Here am I!” and demand attention. Or they negate symptoms. (31, woman) Maybe women are sometimes tougher, and so they are not dialysed as early as men. (39, woman)
Avoiding burden on the family	Women don’t want to burden others with what’s going on with them. There are woman who I will explicitly ask, do your kids know […] […] trying to avoid burdening the family. (04, woman) I’ve certainly heard women more often say things like they don’t want to be a burden on people. (15, woman) They don’t want to be a burden, particularly the older ones. They don’t want to be a burden on their families because they […] realize that it may be labour-intensive. (24, woman)
Isolation and coping alone	Women in dialysis are less cared for […] less support from partners […] not appreciated […] abandoned by the husband because […] not performing as a women, […] (01, man) When I see that the female patient is not in good shape, the husband is not taking care of her correctly or doesn’t believe that she’s so bad. Then I talk to the family. (09, man) Women […] left alone. The husband never comes and they never, with few exceptions, provide supports […] on PD. […] women more depressed because […] really on their own. (18, man)
Stereotyping, stigma, and judgment	
Body image	Women patients, some of them, especially on the younger side, are afraid of de-feminization. That they’re not really women and they’re not attractive any longer. (11, man) For women, it’s harder to have this discussion about weight loss. They obviously feel worse about their bodies in general, they feel more judged if they haven’t lost weight, or gained weight. Women […] more humiliated, more embarrassed, more uncomfortable with these things than the men do. (45, woman) Women may not want a catheter or a fistula for cosmetic reasons. Or they don’t want to be initiated on dialysis because of those reasons like the cosmetic reasons. (51, woman)
Dismissed as anxiety	A woman has had high blood pressure reported for two years and nothing has been done because it’s been attributed to stress. Even the physicians. There may be stereotyping, […] dismissed as psychosomatic. (04, woman) A woman with chronic kidney disease presenting with acute coronary syndrome can just present with maybe extreme anxiety. (36, woman) Women are more often dismissed by cardiology as not having real cardiac disease […] Increasing physician and patient awareness that there may be unconscious biases in the way that people ask questions, or hear the answers […], or contextualized symptoms […] important. (46, woman)
Shame and embarrassment	There is that level of judgment, particularly around fertility issues. In this particular Bhutanese community, your fertility is directly relevant to your value in that society. (24, woman) Sexuality is a topic that often comes up for me. […] Male doctors would never ask a woman if this is a problem. Although men do bring it up with me. But maybe they’d rather talk to a male […]. (31, woman)
Weakness and frailty	Because women are on average smaller than men, sometimes there are assumptions about how sick an older woman may be compared to […] an older man […]. This little lady is too frail to do home therapy […] more likely to be offered palliative care […] look at her as being sicker. (06, woman) Females, particularly older females, probably are assessed more quickly to be frail. (25, woman)
Protecting masculinity	
Safeguarding the provider role	Males want to be dialysis free until they retire. (12, man) Particularly young Polynesian men or minor Pacific men, they’re often trying really hard to be the man of the family and that’s not compatible with being sick, so they can’t be sick. […] (14, man) If I now have a man who has to feed his family, he may pay less attention to his blood pressure, […] than a woman who is not responsible for maintaining the family budget. (33, woman)
Clinging to control	Men, they feel that they lose control. They depend on another person. They don’t like that.(13, woman) The men frequently they disappear for a long time and return very bad. He said his life was not good because he had to go three times a week, […] (17, man) Men think I’m strong and I don’t need help type thing. Especially in the early stages of CKD, until they crash and burn, and end up on dialysis. (19, man) In the lead up to the disease, men are in denial but once they accept their diagnosis, they’re gunning for best treatment options and so they are a bit more focused towards outcome. (24, woman)
Self-regard and entitled	A wife will change her schedule because it’s her husband’s surgery but doesn’t happen as often when the roles are reversed. (04, woman) They think about themselves first before thinking about the greater good of the family. (24, woman) Very often there is a patriarchy. The man says that the woman has to do something. He involves her in his own illness. (31, woman) So whether I treat male patients better? Men are perhaps the more demanding, more tiresome patients who ask questions, who don’t let you off so quickly. (32, man)
Decisional power and ownership	
Male dominance in decision-making	In some cultures, men tend to make the decisions and the women go with it. Often the men will speak for the women even during the consultation. They do all the talking. (09, man) Pacific women are potentially much more deferential. (21, woman) It’s certainly a difference with a male dominated culture. Women will defer to the male person to make decisions around dialysis and getting to a treatment endpoint. (24, woman) There are a lot of European countries included which have a clearly patriarchal system. The husband says his wife can’t go for dialysis three times a week. (34, woman)
Analytical approach	Women are more conservative and more willing to take advice on and more willing potentially, they appear to be more deliberative and collaborative. (21, woman) Most of my women patients will accept their diagnosis earlier but be more hesitant about treatment options. They often take longer to make decisions. (24, woman) Men tend to take a lot as “given” […] guided by our recommendations, […] in contrast to women, who also talk to patients or get information from the Internet or elsewhere. (30, man)
Inequities compounded by social disadvantage	
Financial and transport barriers	Many women do not have a driving licence, they are dependent on neighbourhood help or public transport. (31, woman) Those women who cannot afford childcare and are tied up at home […] are at a disadvantage, especially in the lower social classes. […] money flows more often to the man (31, woman) […] there isn’t really money often for the women to go to the hospital. […] In certain places like Africa and possibly India and whatever there’s definitely systematic disadvantage of women. (45, woman)
Without social security	To have social security, you have to work. It’s more likely that the men are working and therefore have social security than women. So there are delays for women or no [dialysis] therapy. (90, man) […] they might more easily make the decision to not go on dialysis if it’s a female. Whereas, if it’s a male in question, they might really try to raise resources in order to […] provide dialysis. (35, man) Women are less likely to go onto dialysis, but not really because of choice, but because of cost. […] What happens? The outcome is poor because you’re going to die. (37, woman) Men are the ones who tend to show up in hospital because there isn’t really money often for the women to go to the hospital. (45, woman)
Limited literacy	I have to spend a lot of time explaining it, getting an interpreter involved, depending on which country they come from, they can have different perceptions about […] their trust in the system. (16, woman) Those who have no education and no network are left empty-handed and are not getting the medicine. (31, woman) These ladies are busy looking after the children, cleaning the house, doing all these things, [.] don’t have time to integrate themselves into society, […] don’t always understand their disease. (45, woman)
Entrenched discrimination and vulnerability	There is the old, conservative role model, where the man is more important than the woman. That also goes back to the Sharia, where it says that the woman is worth half as much as the man. (31, woman) Women are typically disadvantaged socially, they don’t have, in many societies, an equal social status as men. […] women’s health is often the one that often goes on the chopping block. (35, man) If a family has a precarious situation, they might more easily make the decision to not go on dialysis if it’s a female. If it’s a male […], they might really try to raise resources […] provide dialysis. (35, man)

CKD, chronic kidney disease; ID, identification; PD, peritoneal dialysis.

Number indicates participant ID.

### Primary Commitment to Caregiving

#### Coordinating Care

Participants noted that female caregivers often took responsibility in coordinating access to CKD education sessions and clinics and usually accompanied men to appointments. Women wanted to “get involved in the solution” and decision-making and were “willing to lose some of their own goals and life” to support their partner. Some stated that men had agreed to see a doctor only because their “wife, girlfriend, told me to see a doctor.”

#### Taking Charge of Health Management

Some explained that female caregivers tended to take control of managing the patient’s medications, diet, and home dialysis. Often, men did not know what “pills” to take and depended on their wives who had “more knowledge and control” over medications. Men relied on their wives to do dialysis-related tasks, including cannulation, and would “really fall apart” without the help of their partner. Some stated that some men receiving dialysis “were alive” and “complied with treatment” because of their wives. Women usually did the “cooking and shopping” were therefore responsible for managing their husband’s dietary restrictions. Some recognized that nephrologists directed instructions to wives instead of the husband.

#### Deprioritizing Own Health

Participants were concerned that women missed appointments (e.g., for follow up, creation of vascular access, biopsy) or dialysis sessions because of family responsibilities—”she would miss out on at least once a week dialysis session because she felt that she had to be at home to run the errands and for the young children.” They did not have time for lifestyle management (e.g., exercise). Women were “always prioritizing the role as a caregiver and forget that they need to take care of themselves.” In certain cultural groups, women were expected to be caregivers and to do “what’s right for the family unit.”

#### Centrality of Family in Decision-Making

Participants commented that women made decisions on dialysis based on family responsibilities—”because I have my child, I have my husband, I have my parents to look after, not because I need it for myself.” Women were concerned on the risk of dialysis on pregnancy. Some also stated that women would still pursue pregnancy even if it meant they may “lose their kidneys” faster. Some mothers were motivated to do dialysis because of their children—”to fulfill the nurturing role in their life.” The decisions of women “involved goals that goes beyond them” and participants strived to help “reconcile” dialysis with the family priorities of women, for example, by scheduling dialysis outside of school pick-up times.

### Vigilance and Self-Reliance

#### Diligence and Conscientiousness

Participants believed that women were generally more organized, “careful,” and diligent in adhering to lifestyle changes, medications, and monitoring health (e.g., hypertension and diabetes), particularly in the earlier stages of CKD. Some speculated that women were accustomed to managing multiple demands and had more interaction with the health system (e.g., for pregnancy and gynecologic issues). By comparison, they speculated that men “often work outside, eat bad foods, and drink more,” wanted to “enjoy life,” and were more “stubborn” compared with women.

#### Stoicism in Tolerating Symptoms

Some remarked that women seemed to adapt and have a higher tolerance of symptoms, including fatigue, because of their focus on other responsibilities. Women were said to be less likely to “shout” on their symptoms and “demand attention.” In comparison, participants explained that men were “more likely to say that everything is fine” because they felt unable to “verbalize their pain.”

#### Avoiding Burden on the Family

Participants emphasized that female patients tried to avoid imposing physical and mental burden on their family—”women are trying to spare their family from the burden of their disease.” Women usually did not want others to help with home dialysis, opted for the dialysis modality that was minimally disruptive to the family, and tried to protect their family members from anxiety by refraining from talking with family on dialysis.

#### Isolation and Coping Alone

Some observed that women were inclined to “put up with [CKD] on their own and manage it themselves,” and usually attended appointments alone. They suggested that loneliness and isolation exacerbated depression and anxiety. In some cases, women with CKD were abandoned by their partner because they were “not performing as a woman … she passes to a secondary role, the husband, the kids don’t respect her.” Some participants advised women to identify a care partner or spoke with the family if they sensed that the husband did not take her condition seriously.

### Stereotyping, Stigma, and Judgment

#### Body Image

Some indicated that women held concerns on their appearance and were “afraid of defeminization” and were thus reluctant to have interventions, such as vascular-access creation, which also delayed commencement of dialysis. Discussing weight loss in managing CKD was difficult because women “feel worse about their bodies, they feel more judged if they haven’t lost weight or gained weight” and were “embarrassed” and “humiliated” when they were unable to lose weight.

#### Dismissed as Anxiety

Female participants speculated that nephrologists held “unconscious biases” whereby they dismissed risk factors or signs of CKD in women “as psychosomatic.” In some cases, hypertension in women was “attributed to stress” and was not further investigated and managed. In another example, a female patient with CKD and acute coronary syndrome seemed to present with “extreme anxiety.”

#### Shame and Embarrassment

For some participants, broaching sensitive topics regarding fertility, sexual health, and mental health could be difficult if the patient’s gender was different to theirs—”that is definitely also more of a gender-specific way of communicating to each other and it’s easier with the same gender.” Some women were ashamed to discuss fertility, particularly if their ability to bear children was attached to their “value in that society.”

#### Weakness and Frailty

Some recognized that nephrologists could make “assumptions about how sick an older woman may be” compared with their perception of men because of differences in physique. There was some concern that nephrologists deemed some women to be “too frail to do home therapy” so were more inclined to reduce the dialysis prescription or suggest nondialytic supportive management without explicitly asking women on “functional limitations.”

### Protecting Masculinity

#### Safeguarding the Provider Role

Participants noted that male patients were focused on their work, and being sick with CKD was not “compatible” with being the “man of the family,” and therefore, some struggled to accept their illness. Men missed clinical appointments, delayed commencement of dialysis, or did not monitor their health (e.g., blood pressure) because they were “the breadwinner for the family” with little time to focus on health. They remarked that men generally wanted “to be dialysis free until they retire” and were reluctant to undergo vascular-access surgery because it could interfere with their “heavy labor work.”

#### Clinging to Control

The diagnosis of CKD meant a loss of control over their lives, particularly in male patients. CKD “put a brake” on patients’ lives, and it was apparent that men were often in “denial” because they wanted to remain strong and independent. Some reported that some men would “disappear for a long time and return very bad” and sometimes “quit dialysis sessions” because they wanted freedom. Nevertheless, on accepting their diagnosis, men were “gunning for the best treatment option.”

#### Self-Regard and Entitlement

Participants noticed that in patriarchal cultures, men with CKD expected women (wife, mother) to care for them and were “spoiled by women at home.” Men were inclined to “think about themselves first” and to “involve women in his own illness.” Some remarked that once men accepted their condition, they were more “demanding, louder, blustering” and would therefore receive more attention and better treatment from clinicians compared with women. Some participants tried to foster a sense of ownership of treatment among men, saying to them—”you have to do the therapy, not the partner.”

### Decisional Power and Ownership

#### Male Dominance in Decision-Making

Participants observed that, in certain ethnic or religious contexts, men were upheld as the primary decision-maker whereas female patients were more “deferential” and sought their husband’s decision or approval before agreeing to treatment (e.g., dialysis). Men would “speak for female patients during the consultation” and “dominate” the conversation, which participants believed was “catastrophically bad … you can’t talk to the woman, everything runs via the man … you can’t hear anything from the patient, it’s just filtered.” In one example, a husband did not allow his wife to go for dialysis 3 times a week. Some participants deliberately arranged to speak with the female patient directly and encouraged women to be “empowered” to assert themselves.

#### Analytical Approach

Some indicated that women were more “deliberative and collaborative” when making decisions on treatment (e.g., dialysis) and would take more time to make decisions compared with men. Women with CKD would talk to other patients and seek information from other sources (e.g., from the internet).

### Inequities Compounded by Social Disadvantage

#### Financial and Transport Barriers

Participants were concerned on women from socially and economically disadvantaged backgrounds who faced difficulties in accessing dialysis and medications. In some settings, many women did not have a driver’s license and could not afford transportation or had to rely on neighbors to attend appointments. Women who could not afford childcare were unable to attend clinics and “reached out less” to obtain resources and support.

#### Without Social Security

In countries without universal coverage for dialysis, participants voiced that women with CKD were extremely vulnerable. In India, Mexico, and Africa, for example, families prioritized resources for men because they were the “breadwinners” and men had control of the finances—”if a family has a precarious situation, they might more easily make the decision to not go on dialysis if it’s a female. If it’s a male, they might really try to raise resources to provide dialysis.” Furthermore, men could access social security individually through their employment to cover dialysis—”in Mexico, it’s more likely that the men are working and therefore have social security than women. So there are delays for women or no therapy.” In the United States, women from ethnic minority groups were less likely to be insured and therefore had less access to treatment, including dialysis.

#### Limited Literacy

Some remarked that women with CKD in certain cultural groups or who were immigrants had little capacity to self-advocate and negotiate the system because they were less socially integrated into society, had poor education and health literacy, and encountered language barriers. Participants were frustrated by this “injustice” and “unfairness.” Men had more opportunities to learn language and socially integrate through work whereas women were expected to do household duties “and so remain fairly isolated and then don’t always understand their disease.”

#### Entrenched Discrimination and Vulnerability

Participants felt helpless as they witnessed women with CKD being inherently and profoundly disadvantaged in patriarchal social structures, who were dependent on their husband as the provider. They saw that women felt “insecure” and “vulnerable,” had low self-worth, and believed that they did not deserve to receive care because they did not have “equal social status as men” in societies where “men are prized possession.” In some cultures, participants commented the “woman is worth half as much as the man.” They were deprioritized in the family—”when there are competing priorities in families with limited incomes, then women’s health is often the one that often goes on the chopping block.”

## Conclusion

In this study on perspectives of nephrologists regarding gender disparities in CKD and dialysis, nephrologists perceived that, in general, women strived to fulfill their fundamental role in caring for the family, which extended to caring for their partner with CKD, and this involved coordinating access to care and managing their diet, dialysis, and medication regimen. For women with CKD, they sometimes deprioritized their own health because of family responsibilities and made treatment decisions in consideration of the potential impact on their family. Women were found to be more vigilant and self-reliant to avoid burdening their family; however, this could lead to isolation and depression. Some participants recognized potential unconscious bias among nephrologists in assuming women were more frail, weak, and susceptible to psychological problems (e.g., anxiety), which affected how some nephrologists made treatment decisions. Some believed that men were focused on protecting their masculinity and had a stronger sense of self-regard such that they expected to be supported by women. Men held power in decision-making whereas women were observed to take a more analytical approach to treatment decisions, such as commencing dialysis.

Overall, the themes were identified by both men and women who participated in the study. Additional intersecting challenges were apparent and were related to gender disparities specific to low-resource settings, countries without universal healthcare coverage, and in communities with a patriarchal structure. In low- and middle-income countries or in socioeconomically disadvantaged communities, women with CKD encountered financial barriers in accessing health care. They were unable to afford transport, childcare, or treatment (dialysis and medications) and were reluctant and unable to seek help and resources. In countries without universal coverage, women from ethnic minority groups (e.g., African American women and undocumented immigrants in the United States) were less likely to be insured and were unable to afford kidney replacement therapy. In Mexico, men were more likely to be employed and could access social security through their employer whereas women, who were often at home and did not work, could not readily access dialysis. Women were also found to be particularly vulnerable in patriarchal communities where men held power, worth, and higher status. Families would prioritize men and may be reluctant to mobilize resources for women to receive dialysis. Women inherently felt insecure and inferior, deferred decisions on their care to men, and did not assert or advocate for their own health.

Earlier work indicates that more women have CKD but are disproportionately represented among patients receiving kidney replacement therapy^1^ whereas men have a higher risk of CKD progression^4^^,^^30^ and mortality.^4^^,^^31^ To the best of our understanding, the previous evidence for this inequity has been derived entirely from epidemiologic studies: on sex-specific kidney replacement incidence and prevalence on one hand^28^^,^^29^^,^^32^, ^33^, ^34^ versus CKD prevalence on the other hand^35^, ^36^, ^37^, ^38^, ^39^, ^40^, ^41^, ^42^, ^43^, ^44^, ^45^, ^46^, ^47^, ^48^, ^49^, ^50^, ^51^, ^52^, ^53^, ^54^, ^55^, ^56^, ^57^ (summarized in Carrero *et al.*^1^). As the present analysis is, to our knowledge, the first to have involved qualitative data, it should be interpreted with caution. Nevertheless, our analysis now provides an opportunity to discuss reasons for the sex/gender disparity that could previously not be mentioned in the context of epidemiologic data. Gender-based reasons for the underrepresentation of women in the dialysis population may be that women, during the initial stages of CKD, may be more proactive and vigilant in monitoring their health, thereby perhaps being able to postpone dialysis initiation. By contrast, delays in accessing dialysis may be due to competing priorities of family responsibilities, fear of imposing burden on their family, higher tolerance of symptoms, concerns that interventions may change their appearance, and being more analytical in their decision-making. We acknowledge that some of the reasons may also be biological. In some settings, women may also be socioeconomically disadvantaged and therefore be financially unable to choose dialysis as a treatment option even if they desired it. In the dialysis population, women may feel that they are expected to cope on their own and miss dialysis sessions or appointments because of family commitments, which could contribute to the increased risk of hospitalization and impaired quality of life in women.^5^, ^58^, ^6^, ^7^^,^^58^

We generated detailed and novel data on gender disparities in CKD and dialysis from the perspective of nephrologists. We used investigator triangulation and member feedback to ensure that the findings reflected the full range of data, and we recruited participants until data saturation. Nevertheless, there are some potential limitations. The candidates approached for interview were to a large degree drawn from the professional networks of the investigators. On the basis of our key informant approach, we included some participants who had experience and knowledge on gender difference in CKD. Of note, some participants did not provide perspectives on gender-based disparities in CKD. This concern is supported by Lipford *et al.*^15^ who found that understanding of gender-based disparities was limited among dialysis staff. Further reflection on and discussion of this element would be interesting and may provide useful additional insights in how these inequities can begin to be addressed.

Among additional limitations, it has to be mentioned that relevant insights should also be captured in future studies with women who have CKD. Further insights from diverse socioeconomic regions may also be needed as gender disparities could be more pronounced in low- to middle-income countries. Notably, however, the results of this study were neither unclear nor ambiguous. The clarity of the results also refutes the idea that justification is needed why this work should be an international study, given that gender is also embedded within a cultural context. The interviews were conducted in English and German languages only. Given the scope, we did not included data on disparities in transgender patients with CKD. Following this work, survey will be conducted with patients and clinicians to ascertain the frequency of opinion related to gender disparities in CKD and will also focus on decision-making in particular contexts—such as initiation of dialysis, transplant issues, and self-care.

The need to recognize and address gender disparities in health care is strongly advocated across medical and health disciplines, including nephrology.^11^ Recommended approaches include addressing the structural dimensions of gender inequality (e.g., law and policy reform, increase participation of women in decision-making roles), challenging gender stereotypes, targeting gendered exposures and vulnerabilities (e.g., social security, support for childcare), transforming the gendered politics of health systems (e.g., universal healthcare, training clinicians to apply gender perspectives), improving the evidence base for policies, making organizations more accountable to gender equality and equity, and supporting women’s organizations to support advocacy efforts.^22^^,^^59^^,^^60^ Although gender disparities have unique consequences on access to care and outcomes in CKD and dialysis, they are not well understood and perhaps under-recognized in practice and policy. On the basis of the findings, we have provided suggestions for clinicians in the areas of improving awareness and education, communication, addressing unconscious bias, supporting empowerment in decision-making and self-management, and facilitating access to financial support and health care (Table 3).

**Table 3:** Suggestions for addressing gender disparities in CKD and dialysis

Domain	Suggestions
Empowerment in decision-making and self-management	Engage trained independent interpreters to support direct communication with women (women) Establish treatment regimens that are flexible with and centered on life priorities, for example, caregiving responsibility and work (women, men)
Financial support	Advocate for the provision of universal healthcare coverage/insurance for CKD treatment and dialysis, particularly for those who are socioeconomically disadvantaged Provide assistance in accessing financial support, for example, childcare (women) Work with stakeholder organizations, including government and charity organizations, to establish grants specifically for women (women)
Patient awareness and education	Emphasize and encourage ownership of treatment (men) Identify care partners to provide support (women)
Communication	Address appearance and body image concerns, for example, in relation to vascular access Encourage lifestyle management using a sensitive and positive approach
Unconscious bias in clinicians	Establish system alerts for comorbidities, complications, and laboratory results to avoid dismissing symptoms (women) Conduct explicit and object assessment of capacity and functioning to inform treatment decisions (women)
Access to clinics	Establish and provide outreach or mobile clinics (for dialysis, medical consultation, educational sessions)
Accountability	Establish institutional policies and mechanism for accountability in addressing gender disparities

CKD, chronic kidney disease.

Health systems and clinicians may reinforce traditional gender roles of patients and overlook gender inequalities, and it has been suggested that there is a need to “disrupt gender norms.”^61^ Nephrologists in our study emphasized the need to support women in identifying a care partner and to establish treatment plans in consideration of family commitments. We also suggest creating opportunities wherein women feel safe and empowered to make decisions on treatment and to seek help in obtaining resources they need to manage their health. Furthermore, another approach is to encourage men to take more ownership of CKD and their treatment, including dietary management and home dialysis. Adopting and enacting gender consciousness may help to alleviate societal pressures and barriers for women with CKD, so they can have better access to care and treatment.

Overall, from the perspective of nephrologists, women with CKD faced gender-related barriers in accessing care, which relates to social norms and roles of caregiving and fulfilling family responsibilities, managing and coping alone, and being judged as emotionally and physically weaker. Some women may be even more vulnerable in patriarchal communities where men hold decisional and financial power, status, and social worth, such that women have little capacity or resources to access treatment, including dialysis to survive. Increased understanding and consciousness of these gender disparities emphasize the need to explicitly address social norms, power differences, systemic patriarchy, and unconscious bias, which may improve equitable care and outcomes for all people with CKD.

## Disclosure

All the authors declared no competing interests.

## Supplementary Material

Supplementary File (PDF)

**Supplementary File 1:** Interview guide.
