# Supplementary material for: Nephrologists’ Perspectives on Gender Disparities in CKD and Dialysis
Source: Kidney Int Rep. 2021 Nov 9;7(3):424–35. doi: 10.1016/j.ekir.2021.10.022 (PMC8897691; doi:10.1016/j.ekir.2021.10.022)
Supplement: Supplementary File (PDF) [file mmc1.pdf]

Supplementary File 1. Interview guide

Introduction

We are interested in the role of gender in patients' access to care and outcomes in CKD and dialysis. We would like to hear your thoughts about whether you think there is a role of/or differences in gender related to access to care, treatment decision-making, self-management and outcome; and why.

Gender refers to the roles/responsibilities, activities, characteristics, opportunities that might be considered the "norm" or is appropriate for women or men. **Use this as a reference/prompt across all questions*

Opening

1. Could you tell me briefly about your role in caring for patients with CKD?
2. Have you observed or can you speculate about any differences between men and women in any aspect of their experience of CKD?

Access to care

3. Do you think there are differences relating to the diagnosis of CKD between women and men – why/why not?
4. What do you think about equity of access to care for patients with CKD, are there any differences based on particular characteristics – why? (cultural background, education, financial/employment, other roles, gender) ➔ dialysis/transplant
5. Do you think there are any barriers or challenges that are specific to women regarding access to healthcare, any specific to men? (primary care, nephrology) ➔ dialysis/transplant

Communication and treatment decision-making

6. In general, how do you approach decision-making about commencement of kidney replacement therapy for patients with CKD? What are the things you consider from your perspective? Can you tell me about some of the factors that might make these decisions a bit more complex/difficult? ➔ dialysis/transplant
7. Are there any factors that you considered that are specific to women/men? Do you have any examples?
8. Have there been times when you recommended to start dialysis but your patients did not want to start dialysis – what were the reasons, and any that are gender-specific? How did you negotiate/navigate this? What about for tranmsplant?
9. Some studies have indicated that disproportionately less women commence dialysis/kidney replacement therapy compared with men. **[VISUAL AID]**[1 2] What are your first reactions, how would you explain this? (social support from family/friends, roles and responsibilities, work/study commitments, culture, religion, emotions)
10. Do you think communication between doctors and patients may be different between women/men (discussing sensitive topics)?

Self-management and coping

11. What is your role or approach regarding self-management (patients taking medications, lifestyle, attending appointments) Are there any aspects that might be specific to women/men? ➔ dialysis/transplant
12. Do you think there are gender-related aspects that impact on the patients' motivation or ability for self-management? What are some examples? ➔ dialysis/transplant

Outcomes

13. Do you think there is any disparities in outcomes between women and men regarding outcomes (risk of progression of CKD, mortality, CVD, other outcomes) – why?
14. Do you use any specific strategies for women/men to minimise their risk of complications/comorbidities, and improve outcomes?

Close

Do you have any suggestions for specific ways to improve access to care or outcomes for women/men ➔ dialysis/transplant

Is there anything else you would like to add?

Thank you.

1. Carrero JJ, Hecking M, Chesnaye NC, Jager KJ. Sex and gender disparities in the epidemiology and outcomes of chronic kidney disease. *Nat Rev Nephrol* 2018;14(3):151-64.

2. Hecking M, Bieber BA, Ethier J, Kautzky-Willer A, Sunder-Plassmann G, Säemann MD, et al. Sex-specific differences in hemodialysis prevalence and practices and the male-to-female mortality rate: the Dialysis Outcomes and Practice Patterns Study (DOPPS). *PLoS Med* 2014;11(10):e1001750.
